# Positive-bias gate-controlled metal–insulator transition in ultrathin VO_2_ channels with TiO_2_ gate dielectrics

**DOI:** 10.1038/ncomms10104

**Published:** 2015-12-14

**Authors:** Takeaki Yajima, Tomonori Nishimura, Akira Toriumi

**Affiliations:** 1 Department of Materials Engineering, The University of Tokyo, Tokyo 113-8656, Japan; 2 JST-CREST, Tokyo 113-8656, Japan

## Abstract

The next generation of electronics is likely to incorporate various functional materials, including those exhibiting ferroelectricity, ferromagnetism and metal–insulator transitions. Metal–insulator transitions can be controlled by electron doping, and so incorporating such a material in transistor channels will enable us to significantly modulate transistor current. However, such gate-controlled metal–insulator transitions have been challenging because of the limited number of electrons accumulated by gate dielectrics, or possible electrochemical reaction in ionic liquid gate. Here we achieve a positive-bias gate-controlled metal–insulator transition near the transition temperature. A significant number of electrons were accumulated via a high-permittivity TiO_2_ gate dielectric with subnanometre equivalent oxide thickness in the inverse-Schottky-gate geometry. An abrupt transition in the VO_2_ channel is further exploited, leading to a significant current modulation far beyond the capacitive coupling. This solid-state operation enables us to discuss the electrostatic mechanism as well as the collective nature of gate-controlled metal–insulator transitions, paving the pathway for developing functional field effect transistors.

## Abstract (web summary)

Oxide materials that undergo a metal to insulator transition under an applied electric field could extend the functionality of electronic devices. Here, the authors induce a phase transition in vanadium dioxide via a titanium dioxide gate, and thus modulate the current flowing through the channel.

## Graphical abstract

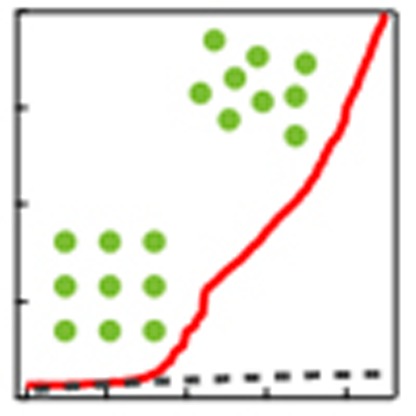

Metal–insulator transitions (MITs) in transition metal oxides often introduce nontrivial changes in a wide range of electrical, magnetic and optical properties^1^. Therefore, the MITs can incorporate various functionalities in materials themselves rather than in the device structures^2^, further boosting the development of electronics in conjunction with silicon (Si) technology. The MIT originates from electrons in narrow *d*-orbit bands, which are often localized by electron–electron and electron–phonon interactions^1^. These interactions can be weakened by external stimuli such as temperature change and electron doping. Then, all the localized electrons are delocalized, making a sudden change from low to high electrical conductivity, namely the MIT.

The dynamic control of the doping-induced MIT has been attempted via electrostatic gating in cuprates and manganites^3^,^4^,^5^,^6^ for the purpose of gate-controlled MITs. Although the gate-controlled MITs have partially been successful, the expected large conductivity modulation has been hindered by the gradual transitions during the MIT due to the coexisting metallic and insulating domains^4^,^6^. On the other hand, a larger conductivity modulation is expected by MITs in vanadium dioxides (VO_2_; ref. 7) because VO_2_ shows a relatively abrupt transition in thin films^8^,^9^,^10^, and even a completely discontinuous transition in free-standing nanobeams^11^. In addition, VO_2_ has several industrial advantages: a simple composition, a relatively low temperature of 300 ^o^C for growing a single-crystalline film on TiO_2_ (refs 8, 9, 10) and a transition temperature higher than the room temperature^12^.

The gate-controlled MIT in VO_2_ has been studied for more than a decade. At first, the three-terminal devices with VO_2_ channels were fabricated with the conventional gate dielectrics such as SiO_2_, HfO_2_ and parylene^13^,^14^,^15^,^16^,^17^. However, these devices did not show properties simply expected for the gate-controlled MIT. For example, in a three-terminal device using SiO_2_, the influence of the negative gate voltage (*V*_G_) was observed, but not in a monotonic way^14^. Another example showed current modulation by both positive and negative *V*_G_, where only several % conductivity modulation took more than a few tens of seconds indicating the influence of interface traps^15^. In a HfO_2_ gate device, the current modulation was accompanied by an unexpected hysteresis, which was attributed to the field-induced strain effect^16^. Although the parylene gate showed a clear modulation of infrared ray absorption by negative *V*_G_ and a negligible modulation by positive *V*_G_, the quantitative information was limited^17^. One of the challenges in these previous studies is the limited number of electronic charges accumulated by the conventional gate dielectrics, in the order of 1 × 10^13 ^cm^−2^. In this sense, the gate-controlled MITs in VO_2_ have made a conspicuous development with an emergence of the ionic liquid gate^18^,^19^,^20^,^21^,^22^,^23^,^24^. The ionic liquid gate utilizes the electrical double layer at the solid–liquid interface to accumulate electrons in the transistor channel by an order of magnitude larger density than those achieved by the conventional gate dielectrics. Furthermore, the ionic liquid gate has reported a novel mechanism of gate-controlled MITs in the VO_2_ channel: while the *V*_G_ accumulates electrons only within the electrostatic screening length from the interface, the MIT is induced far beyond this screening length^19^. However, this mechanism has rather been controversial because of the possible electrochemical reaction in the ionic liquid gate, which may induce MITs by forming oxygen vacancy or interstitial hydrogen throughout the VO_2_ channel^20^,^21^,^24^. Besides, the ionic liquid gate has a critical disadvantage of a slow response^22^, severely limiting its device application. To elucidate the mechanism of gate-controlled MITs and to validate their practical interest, the solid-state gate control of the MIT is strongly desired.

In this work, at temperatures close to the MIT, we achieved the gate-controlled MIT by accumulating a significant number of electrons via high-permittivity titanium dioxide (TiO_2_) gate dielectric, realizing current modulation far beyond capacitive coupling. The solid-state device operation not only enables us to discuss the electrostatic mechanism and the collective nature of gate-controlled MITs but also implies the critical role of interfacial structural compatibility: the gate-controlled MIT is possible only for such an interface that all the interfacial atoms can be involved in the structural transformation during the MIT. These results underscore the interaction of the accumulated electrons at the interface with the three-dimensional phase transition, paving the pathway for realizing functional transistors based on the MITs.

## Results

### Inverse-Schottky gate

**Figure 1:**
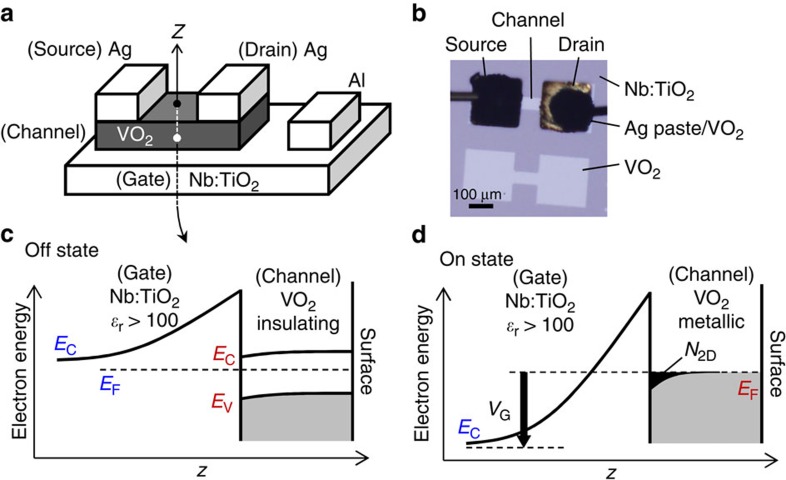
Device structures. (**a**) A schematic illustration, (**b**) an optical micrograph and (**c**,**d**) the band diagrams of the fabricated transistor with the inverse-Schottky-gate structure of VO_2_ (channel)/Nb:TiO_2_ (gate). In **b**, 200 μm^2^ Au blocks were attached to the VO_2_ pads with Ag paste in between for the source and drain electrodes, and the Au blocks were wired with Ag past on the top. The VO_2_ pattern before placing Au blocks is also shown in **b**. The length and width of the VO_2_ channel were 155 and 50 μm, which were measured from the position of Ag paste by removing the Au blocks after measurements. (**c**,**d**) The off state and the on state of the transistor, respectively. In **d**, the electrons accumulated at the interface (black shadow) induce the MIT throughout the whole 6-nm VO_2_ film. In **c**,**d**, *E*_C_, *E*_V_ and *E*_F_ indicate the conduction band edge, the valence band edge and the Fermi level, and *ɛ*_r_, *N*_2D_ and *V*_G_ denote the relative permittivity, the sheet density of accumulated electrons and the gate voltage, respectively.

We focused on TiO_2_ gate dielectric, which has a significantly high relative permittivity above 100 (ref. 25). Given this permittivity, even a relatively thick TiO_2_ gate dielectric (several tens nanometres) can achieve a subnanometre equivalent oxide thickness. In Si technology, the TiO_2_ gate dielectric has not been used because of its insufficient band offset against Si conduction band edge. On the other hand, it can have a sufficient band offset against VO_2_ that has larger electron affinity^26^. A single-crystalline niobium-doped TiO_2_ (Nb:TiO_2_), an *N*-type semiconductor, is used as a back-gate electrode as shown in Fig. 1a,b to realize the inverse-Schottky-gate geometry as shown in Fig. 1c,d. In the terminology of ‘inverse-Schottky gate', the VO_2_ channel is regarded as a metal electrode because of the nanoscale electrostatic screening length of VO_2_ in both the metallic and the insulating phases^21^. In this inverse-Schottky-gate geometry, the depleted single-crystalline Nb:TiO_2_ at the interface can be exploited as a gate dielectric, enabling a high permittivity as well as high breakdown voltage. In other words, the inverse-Schottky-gate geometry achieves electrostatic gating with the aid of strong interfacial electric field created by semiconductor space charge, which was also exploited in other oxide devices^5^,^27^,^28^. For the device operation, the Nb:TiO_2_ gate dielectric accumulates electrons in the initially insulating VO_2_ channel (Fig. 1c) and stabilizes the metallic phase when the accumulated electron density exceeds the threshold as indicated by the black shadow in Fig. 1d.

**Figure 2:**
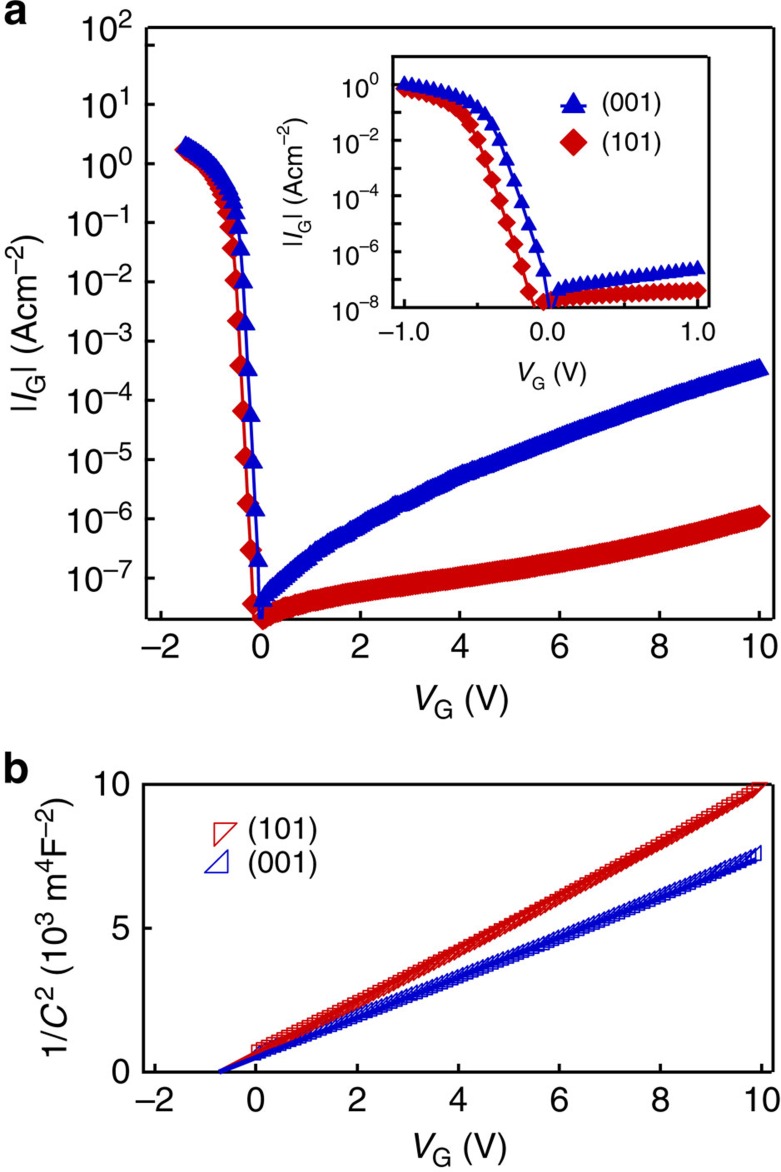
Electrical properties of inverse-Schottky-gate junctions. (**a**) Current–voltage and (**b**) capacitance–voltage characteristics of the VO_2_/Nb:TiO_2_ junctions for (101)- and (001)-oriented interfaces. The inset in **a** shows the magnified current–voltage plots between −1 V and 1 V.

Furthermore, Nb:TiO_2_ has the same crystalline structure of rutile as VO_2_, and, hence, an epitaxial interface is formed in between^8^,^9^. The advantage of this epitaxial interface can be seen in the ideal characteristics of this inverse-Schottky-gate junction as shown in Fig. 2a. The junctions with (101)- and (001)-oriented Nb:TiO_2_ had current–voltage characteristics with a clear rectification at room temperature. The exponential relationship for the negative *V*_G_ (forward bias of the Schottky junctions) followed the thermionic emission model with the ideality factor below 1.1. No dielectric breakdown was observed up to *V*_G_=10 V. Capacitance–voltage (*C–V*) characteristics in Fig. 2b also showed linear relationships between 1/*C*^2^ and *V* (Mott–Schottky plot) for both (101) and (001) orientations. The relative permittivities are estimated to be 111 and 146 for (101)- and (001)-oriented Nb:TiO_2_, respectively, from the slopes in Fig. 2b, which are approximately consistent with the past literatures^25^. The built-in potentials of 0.7 eV were extracted from the intercepts of the extrapolated lines at the voltage axis in both cases, which approximately equals the value estimated from the work functions^26^,^29^. The calculated depletion widths with no bias are 25 and 29 nm for (101)- and (001)-oriented Nb:TiO_2_, respectively, corresponding to equivalent oxide thicknesses of 0.9 and 0.8 nm in the case of metal–insulator-semiconductor structures. Although the decrease in TiO_2_ permittivity under high electric field has been reported^30^, the plots of 1/*C*^2^ are almost linear to *V*_G_ in Fig. 2b, indicating that the decrease in permittivity is insignificant at the electric field of 2.1 × 10^6 ^V cm^−1^ for (101) orientation and 1.8 × 10^6 ^V cm^−1^ for (001) orientation (both corresponding to *V*_G_=10 V). This high permittivity up to high electric field enables electrons more than 1 × 10^14 ^cm ^2^ to accumulate at the VO_2_/Nb:TiO_2_ interface.

### Transistor

**Figure 3:**
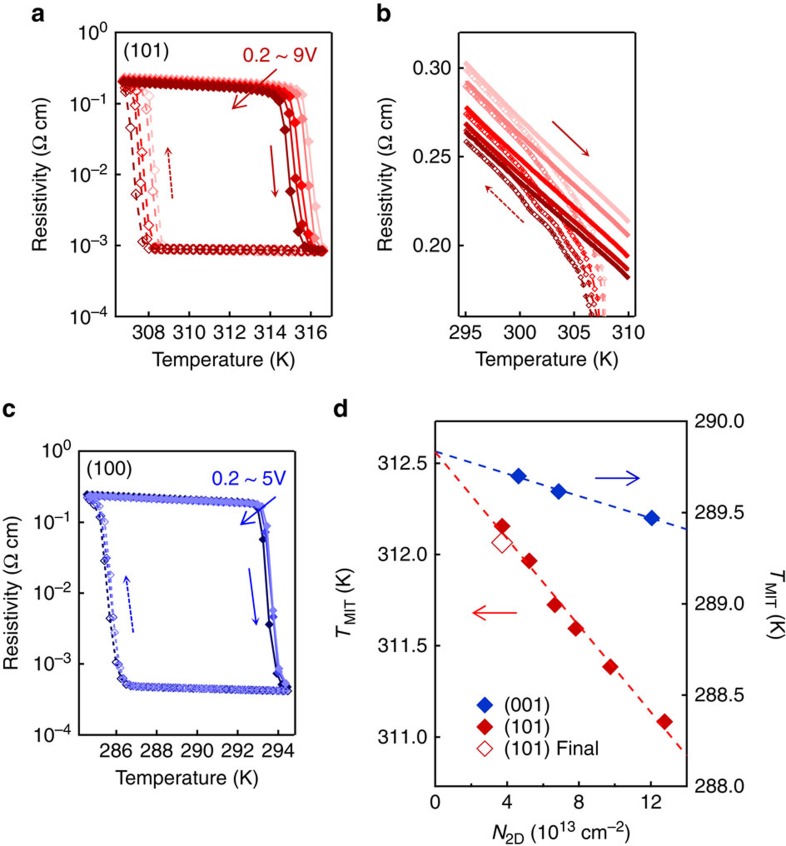
Transistor operation. (**a**,**c**) Resistivity versus temperature for 6-nm VO_2_ films on **a**, (101)- and **c**, (001)-oriented Nb:TiO_2_ substrates. The resistivity was measured for the fixed *V*_D_ of 0.2 V and the *V*_G_ was varied from 0.2, 1, 3, 5 to 9 V for the (101) orientation, and from 0.2, 1.2, 3 to 5 V for the (001) orientation. (**b**) The magnified plot of **a** shows the *V*_G_ effect on the insulating VO_2_. (**d**) Transition temperatures (averaged values for the heating and cooling sweeps in **a**,**c**) as a function of sheet electron density (*N*_2D_) for the (101)- and (001)-oriented interfaces. The measurement was performed from low to high *N*_2D_ (filled diamonds from left to right), and, subsequently, the data of the red empty diamond were taken.

By exploiting these ideal characteristics of the inverse-Schottky-gate geometry, the gate-controlled MIT was investigated on the VO_2_ channel with 6-nm thickness. Figure 3a shows the electrical resistivity versus temperature of the VO_2_ channel deposited on the (101)-oriented Nb:TiO_2_ gate (the lattice mismatch ∼0.8% in the [010] direction and ∼1.6% in the [10–1] direction) as a function of *V*_G_. The data show the MIT with the resistivity jump by more than two orders of magnitude around 312 K, accompanied by a hysteresis reflecting the first-order phase transition of VO_2_ (ref. 7). Remarkably, the MIT was shifted to a lower temperature by as much as 1 K with the application of *V*_G_=9 V. This magnitude of modulation is approximately comparable to the magnitude reported in the ionic liquid gating^18^ (0.5 K for accumulated electron density ∼2.5 × 10^14 ^cm^−2^), which seems to avoid the electrochemical reaction by strictly limiting the magnitude of gate voltage. In the case of the (001)-oriented Nb:TiO_2_ (the lattice mismatch ∼0.8%), on the other hand, the MIT was not clearly shifted by the application of *V*_G_=5 V (Fig. 3c). In the (001) orientation, the MIT was observed around 290 K, which was different from the transition temperature (*T*_MIT_) for the (101)-oriented interface because of the orientation-dependent strain in the VO_2_ channel by the Nb:TiO_2_ substrate^31^. *T*_MIT_ was summarized as a function of the accumulated electron density (*N*_2D_) in Fig. 3d. Here *T*_MIT_ was calculated by averaging the transition temperatures during heating and cooling in Fig. 3a,c. *T*_MIT_ decreased almost linearly to *N*_2D_, and the slope was much larger for the (101) orientation than the (001) orientation.

**Figure 4:**
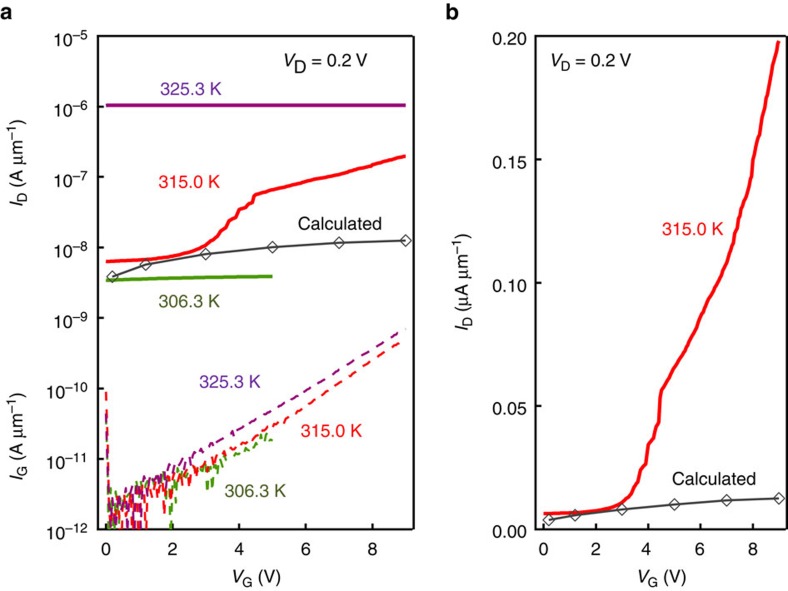
Transfer characteristics. (**a**) Transfer characteristics of the VO_2_ (6 nm)/Nb:TiO_2_(101) transistor with the fixed *V*_D_ of 0.2 V. The four plots in the upper half correspond to *I*_D_ for three different temperatures and the calculated *I*_D_, and the dashed curves in the lower half show *I*_G_. The calculated *I*_D_ was based on the accumulated electron density and the Hall mobility ∼0.5 cm^2 ^V^−1 ^s^−1^ in ref. 31. (**b**) Transfer characteristics at 315.0 K in the linear scale.

Next, the gate-controlled MIT in the VO_2_ channel on (101)-oriented Nb:TiO_2_ was evaluated at three different temperatures: 306.3 K (in the insulating phase), 315.0 K (around the transition temperature) and 325.3 K (in the metallic phase), in the transfer characteristics of Fig. 4a. The channel current (*I*_D_) and the gate leakage current (*I*_G_) were plotted as a function of *V*_G_. *I*_G_ was more than two or three orders of magnitude smaller than *I*_D_ throughout the measurement (except for *V*_G_<0.2 V where the Schottky diode in the gate is forward biased near the drain electrode). The current modulation by *V*_G_ was small at 306.3 and 325.3 K, where VO_2_ remains to be in the insulating and metallic phases, respectively. On the other hand, a large current modulation was obtained at 315.0 K, where *I*_D_ increased nonlinearly with *V*_G_. Besides, the current modulation reached nearly two orders of magnitude by changing *V*_G_ from 0 to 9 V. This magnitude of modulation is much larger than the calculated magnitude as shown by empty diamonds in Fig. 4a (‘calculated'), where the reported Hall mobility of 0.5 cm^2^V^−1^s^−1^, (approximately the same for both metallic and insulating phases; ref. 32) is simply applied to the mobility of the electrons accumulated by *V*_G_. This large discrepancy of experimental values from calculated ones is more pronounced in the linear plot in Fig. 4b, demonstrating the current modulation far beyond capacitive coupling. It should be noted that the actual electron mobility could deviate from 0.5 cm^2 ^V^−1 ^s^−1^, especially when the VO_2_ becomes metallic, given the uncertainty of the Hall measurement in the metallic phase^32^. However, if we can assume that the Hall measurement is relatively valid in the insulating phase, the calculation in Fig. 4a,b may still hold because it basically calculates the electron accumulation in the insulating phase.

### Origin of gate-controlled MITs

**Figure 5:**
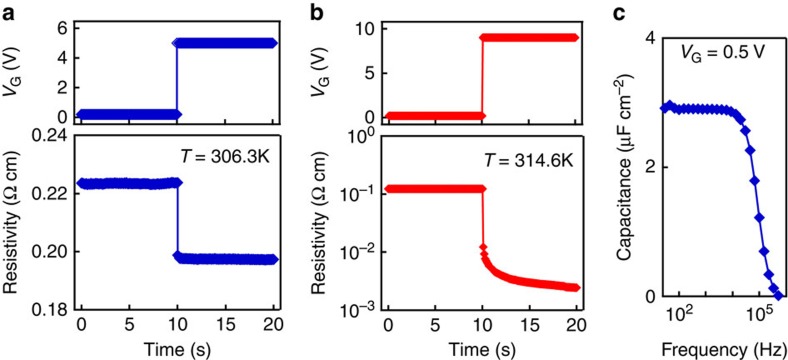
Time response. (**a**,**b**) *V*_G_ and the corresponding resistivity of the 6-nm VO_2_ channel as a function of time for (**a**) 306.3 K and (**b**) 314.6 K. (**c**) The gate capacitance versus measurement frequency at room temperature (insulating VO_2_). For this frequency measurement, the whole VO_2_ film was covered with Au to minimize the influence of series resistance in the VO_2_ channel.

We checked the time response of the channel resistance to *V*_G_ to confirm that the slow process such as electronic trapping and ionic diffusion is not dominant. To investigate the possible influence of these slow processes independently of the MIT in VO_2_, the time response was measured far below *T*_MIT_, where VO_2_ remains in the insulating phase and the application of *V*_G_=5 V decreases the resistivity only by ∼10% as shown in Fig. 3b. When *V*_G_ was abruptly increased from 0.2 to 5 V as shown in Fig. 5a, the resistivity change was faster than the time resolution of this measurement (100 ms). These results indicate that there is neither electronic trapping nor ionic diffusion slower than 100 ms. The fast response was also confirmed by the frequency dependence of capacitance at VO_2_/Nb:TiO_2_(101) Schottky junction far below *T*_MIT_, where the VO_2_ was in the insulating phase (Fig. 5c). The capacitance was approximately constant below 10 kHz, indicating that the capacitance measurement was free from the process slower than 100 μs. Above 10 kHz, the measured capacitance rapidly decreases because of the effect of series resistance in the electrical circuit.

The absence of ionic diffusion in our devices was also confirmed in Fig. 3d. The transition temperature recovered when *V*_G_ was changed from 0.2 to 9 V (red filled diamonds from left to right in Fig. 3d), and finally set to 0.2 V again (red empty diamond). This volatile effect of *V*_G_ indicates the absence of electrochemical reactions, in contrast to the ionic liquid gate where the recovery of the initial state requires an additional process of heating^24^ or applying the opposite *V*_G_ even outside the VO_2_ hysteresis^19^. It should also be noted that the switching power and speed in our device are physically consistent as shown in Supplementary Fig. 1 and Supplementary Note 1, which maps various limits on the power–speed relationship in the VO_2_ channel field effect transistors.

### Character of gate-controlled MITs

One of the issues on the gate-controlled MIT is whether it is different from the temperature-induced MIT in its character. First, we consider the possibility that the gate-controlled MIT is different from the temperature-induced MIT and is determined only by electron density as assumed in the past literature^13^,^14^,^20^,^22^. In this case, the accumulated electron density in our gate-controlled MIT can be compared with critical electron densities for MITs in several other experiments. In our device, *N*_2D_ to decrease *T*_MIT_ by 1 K is ∼9.0 × 10^13 ^cm^−2^. On the other hand, the electron density to decrease *T*_MIT_ by 1 K in W-doped VO_2_ is ∼6.8 × 10^20 ^cm^−3^ (ref. 33), corresponding to ∼1.4 × 10^13 ^cm^−2^ in a 6-nm VO_2_ film. Considering that W doping can also affect crystal structure and hinder a fair comparison, we also focused on the Hall measurement in non-doped VO_2_ films^34^, assuming that the transition is induced when the electron density reaches ∼3.2 × 10^19 ^cm^−3^ in the insulating phase. On the basis of this assumption, the temperature dependence of the Hall electron density indicates that ∼5.0 × 10^18 ^cm^−3^ is needed to decrease *T*_MIT_ by 1 K, which corresponds to ∼3.0 × 10^12 ^cm^−2^ in a 6-nm VO_2_ film. In either case, the electron density that is needed for the gate-controlled MIT is estimated to be only 3–16% of the *N*_2D_ in our device. This discrepancy seems to be not compatible with the assumption that the gate-controlled MIT is driven only by carrier density. In reality, however, it is also possible that not all the accumulated electrons contribute to the transition because of the VO_2_–TiO_2_ interdiffusion on the nanoscale^35^.

Second, we summarize another possibility that the gate-controlled MIT is similar to the temperature-induced MIT, which is known to be a first-order transition mainly balancing the phonon entropy and the orbital energy^36^. In this case, *V*_G_ only modifies the transition temperature of the temperature-induced MIT, and when the modified transition temperature crosses the measurement temperature, the gate-controlled MIT is observed. This possibility was hinted by the slow time response (∼10 s) around *T*_MIT_ (Fig. 5b), where *V*_G_ induces transition from the insulating phase to metallic phase. A possible interpretation of this slow response would be the nucleation of metallic phase domains, which is usually observed in the temperature-induced MIT^37^,^38^. Actually, the nucleation of the metallic phase was also implied by the small but abrupt steps in resistivity in Supplementary Fig. 2 and Supplementary Note 2. Besides, the first-order nature of the gate-controlled MIT was also suggested by the fact that the critical *N*_2D_ (or *V*_G_) for the MIT increases as the temperature decreases from *T*_MIT_ (Fig. 3d). This strong temperature dependence is reasonable if the metallic phase has a higher entropy than the insulating phase, and hence less favoured at lower temperature, just as reported in temperature-induced MIT^36^. At this moment, it is not feasible to determine the character of the gate-controlled MIT, and is to be further investigated via structural or optical analyses, which are beyond the scope of this paper.

### Territory of gate-controlled MITs

In our devices, the current modulation is taking place not only in the vicinity of the interface, but in the whole 6-nm VO_2_ film. This is clearly evinced by the fact that the resistivity versus temperature curve in Fig. 3a was shifted by *V*_G_ almost in parallel without any change in its shape. Given the screening length of VO_2_ is at most a few nanometres^22^, it is implied that the current modulation occurs beyond the electrostatic screening length. The current modulation beyond the electrostatic screening length was also previously reported in the VO_2_ film with ionic liquid gate^19^. However, this current modulation with ionic liquid gate is controversial because of the possible ionic diffusion^20^,^21^,^24^, leaving uncertainty about its origin. In our devices, the solid-state current modulation using the epitaxial VO_2_/Nb:TiO_2_ interface avoids the ionic diffusion as mentioned previously, providing better insights into the collective nature of MITs.

**Figure 6:**
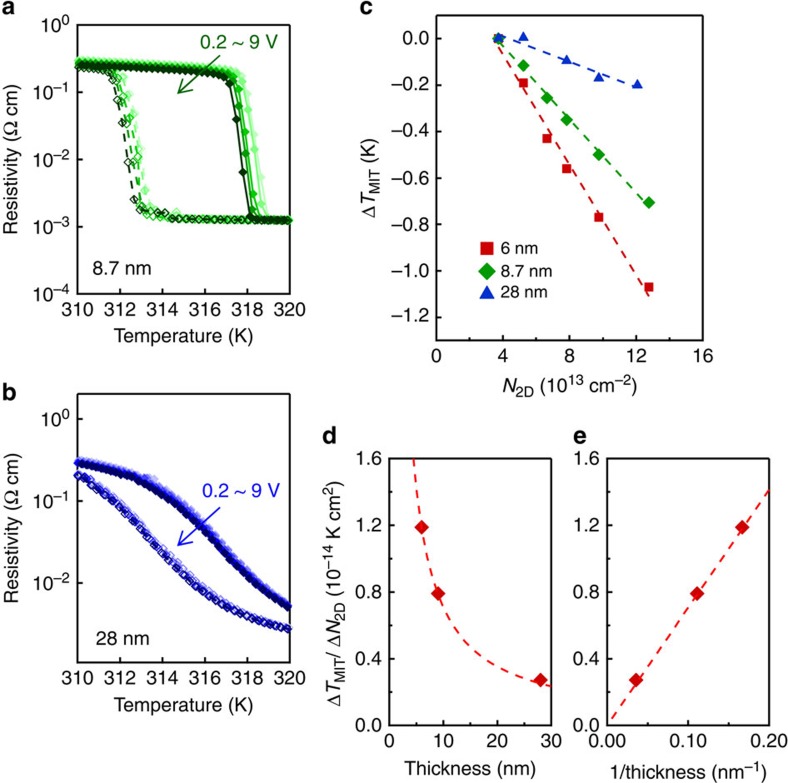
Thickness dependence. (**a**,**b**) Resistivity versus temperature for (**a**) 8.7-nm and (**b**) 28-nm VO_2_ channels on the (101)-oriented Nb:TiO_2_ substrate. The measurement was performed for the fixed *V*_D_ of 0.2 V, and the *V*_G_ was varied from 0.2, 1, 3, 5 to 9 V. (**c**) The change of *T*_MIT_ (Δ*T*_MIT_), which was averaged between heating and cooling, was plotted as a function of *N*_2D_ for three different VO_2_ thicknesses. (**d**,**e**) The average slopes in **c** (Δ*T*_MIT_/Δ*N*_2D_) were plotted as a function of (**d**) VO_2_ thickness and (**e**) its inverse. The dashed line in **e** is a linear fitting, and the dashed curve in **d** is the curve corresponding to this linear fitting.

The current modulation throughout the VO_2_ film was further corroborated by the VO_2_ thickness dependence. Figure 6a,b shows the *V*_G_ dependence of resistivity for VO_2_ thicknesses of 8.7 and 28 nm, respectively. In Fig. 6c, the change of *T*_MIT_ (Δ*T*_MIT_) is plotted as a function of *N*_2D_ for three different VO_2_ thicknesses. As the VO_2_ thickness increases, the magnitude of gate control becomes smaller. Here the slope (Δ*T*_MIT_/Δ*N*_2D_) is a good measure of the magnitude of gate control and was plotted as a function of VO_2_ thickness (Fig. 6d) and its inverse (Fig. 6e). As shown in Fig. 6e, the magnitude of gate control linearly scales with the inverse thickness of the VO_2_ channel, showing that the accumulated electrons interact not only with the interface region but also with the whole VO_2_ film.

The origin of this collective nature could be related to the domain boundary energy between metallic and insulating phases in VO_2_ films. It is well established in the conventional phase transition that small nuclei of phase are unstable because of the energy cost of the phase domain boundary. If we could apply this concept to the MIT in our device, the nucleation of metallic phase only in the vicinity of the interface would be suppressed because it leads to a large area of phase domain boundary inside the film. Instead, the energetically favourable nucleation involving larger volume may occur. Then apparently, the electrostatic effect beyond the screening length could be observed.

### Effect of Joule heating

**Figure 7:**
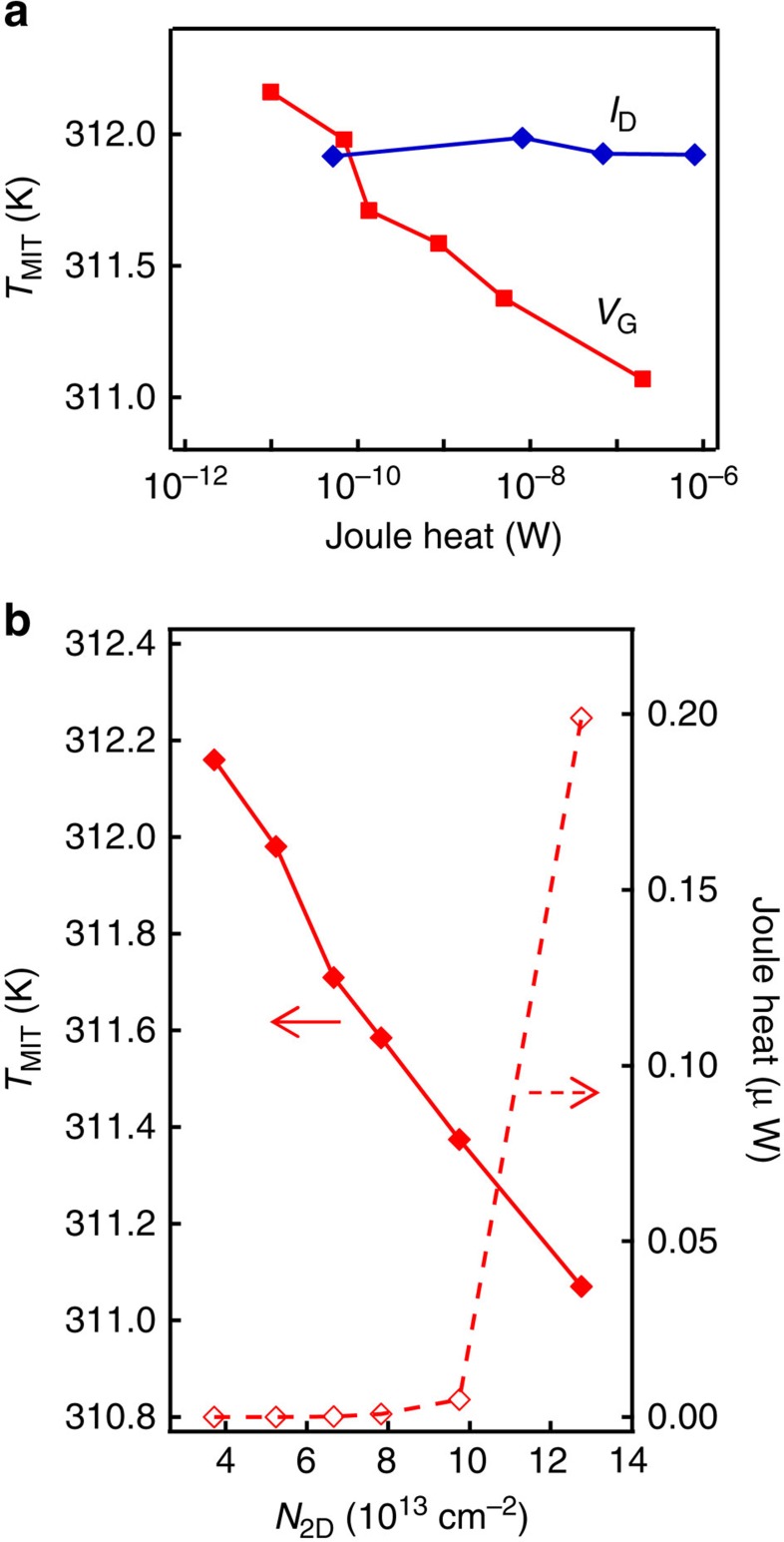
Influence of Joule heating. (**a**) The *T*_MIT_, which was averaged between heating and cooling, was plotted as a function of Joule heat in the VO_2_ (6 nm)/Nb:TiO_2_(101) transistor. For the data ‘*I*_D_', the gate was float and *I*_D_ was set to four different values (0.01, 0.1, 0.3 and 1 μA), where the Joule heat was mainly generated by *I*_D_ × *V*_D_. For the data ‘*V*_G_', *V*_D_ was fixed to 0.2 V and *V*_G_ was set to six different values (0.2, 1, 2, 3, 5 and 9 V), where the Joule heat is ∼*I*_G_ × *V*_G_. (**b**) *T*_MIT_ and Joule heat as functions of *N*_2D_, where *V*_G_ was varied from 0.2 to 9 V.

Several device characteristics indicate that the Joule heating seems to have a negligible influence on the gate-controlled MIT. To check this, we considered the influence of Joule heating in two different ways. First, *I*_D_ was increased (0.01, 0.1, 0.3 and 1 μA) with the gate floated, where the Joule heat was generated mainly by *V*_D_ × *I*_D_. As shown by blue diamonds in Fig. 7a, *T*_MIT_ showed almost no change as a function of Joule heat that was calculated for the insulating VO_2_ around the transition temperature. Second, *V*_G_ was increased with constant *V*_D_, where Joule heat was mainly generated by the gate leakage and was calculated by *V*_G_ × *I*_G_ for the insulating VO_2_ around the transition temperature. In this case, *T*_MIT_ monotonically decreased as shown in Fig. 3d and also by red squares in Fig. 7a. This discrepancy between two plots in Fig. 7a shows that the *V*_G_ dependence of *T*_MIT_ cannot simply be attributed to Joule heating. For the second case, we further compared *T*_MIT_ and Joule heat (*V*_G_ × *I*_G_) as functions of *N*_2D_ in Fig. 7b. While the decrease in *T*_MIT_ is linear to *N*_2D_, the Joule heat is strongly nonlinear, indicating that the decrease in *T*_MIT_ seems not directly related to Joule heat.

While we have shown the Joule heating by homogeneous gate leakage is irrelevant of the gate-controlled MIT, it also has to be considered that the gate leakage current is inhomogeneous, causing local temperature rise (hotspot) in the VO_2_ film. The formation of hotspots can thermally induce local transition from the insulating phase to the metallic phase. Although the total power of this local Joule heating was shown to be not enough to induce transition of the whole film, the metallic phase in the local hotspot can propagate to the rest of the film if it is in a superheated (metastable) state. Thus, the local Joule heating may also account for the observed gate-controlled MIT (Point ‘A' in Supplementary Fig. 3a). The scenario of local Joule heating is also compatible with the slow time response in Fig. 5b as well as the collective transitions in Fig. 6.

Considering the device characteristics in details, however, we can possibly distinguish the influence of local Joule heating (Supplementary Note 3). While the local Joule heating may trigger the transition in the whole film on the high-temperature side of hysteresis (Point ‘A' in Supplementary Fig. 3a) because of superheating (Supplementary Fig. 3b), the same mechanism does not apply to the low-temperature side. On the low-temperature side of hysteresis, the metallic phase in the hotspot cannot stabilize metallic phase in the rest of the film (Point ‘B' in Supplementary Fig. 3c) because the rest of the film is not superheated (Supplementary Fig. 3d), in contrast to the high-temperature side of hysteresis (Supplementary Fig. 3b). In other words, because the insulating phase is more stable than the metallic phase on the low-temperature side of hysteresis, the propagation of metallic phase is energetically forbidden. As a result, the local Joule heating should lead to an asymmetric influence on hysteresis between the high-temperature side and the low-temperature side (Supplementary Fig. 3c), which is inconsistent with the symmetric influence of *V*_G_ in Fig. 3a or Supplementary Fig. 3a. Thus, our device characteristics seem to be not explained only by Joule heating in any way, although we have to keep an open mind considering the long-standing problem of Joule heating in voltage-induced MITs in two-terminal VO_2_ devices^39^,^40^.

## Discussion

While we experimentally showed that *V*_G_ induces the MIT in the whole VO_2_ channel through electrostatically accumulating electrons, the specific mechanisms of the interaction between the accumulated electrons and the whole-VO_2_ channel are yet to be investigated. For example, while the accumulated electrons are expected to be in the conduction band of the insulating VO_2_, another possibility is that they are rather in the in-gap states and only some of them are thermally excited to the conduction band to induce the MIT. We can also consider the influence of inhomogeneity between the metallic and insulating phases during the MIT, which has been observed in polycrystalline^41^,^42^ and single-crystalline VO_2_ films^43^,^44^. The inhomogeneity of different phases is known to cause percolative conduction and sometimes enhance the magnitude of gate control in manganite-channel field effect transistors^4^,^6^.

**Figure 8:**
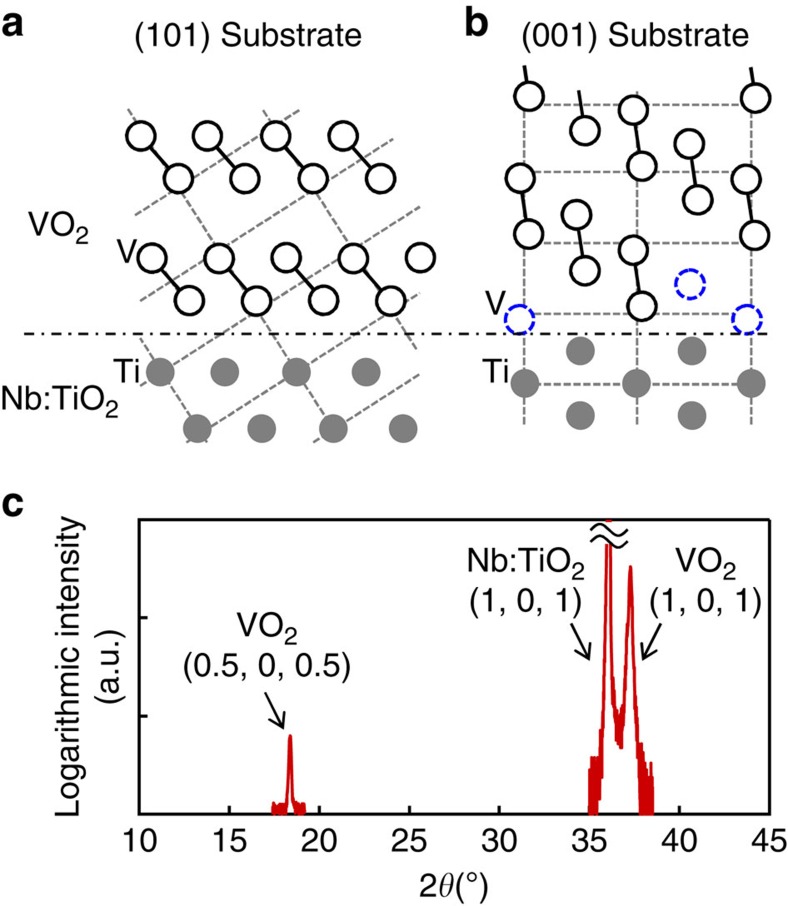
Orientation-dependent interface structures. (**a**,**b**) Schematic illustrations of VO_2_ lattices on (**a**) (101)- and (**b**) (001)-oriented Nb:TiO_2_ substrates viewed in the [010] direction. The dimerized and non-dimerized vanadium atoms are indicated by solid and dashed circles, respectively, while oxygen atoms are omitted. The lattice mismatch is ∼0.8% [010] and ∼1.6% [10–1] in rutile index for the (101) orientation, and ∼0.8% for the (001) orientation. The chain line indicates the position of VO_2_/Nb:TiO_2_ interfaces. (**c**) *θ*–2*θ* scan of X-ray diffraction for VO_2_(71 nm)/Nb:TiO_2_(101) at room temperature, where VO_2_ is in the insulating phase.

The specific mechanism of device operation could also relate to the larger current modulation for the (101)-oriented interface than the (001)-oriented interface. One of the possible origins for this interfacial orientation dependence can be the structural difference between the (101) and (001) interfaces. The (101) and (001) interfacial structures are schematically illustrated in Fig. 8a,b, where the rutile structure is indicated by the dashed lines. The rutile structure of the metallic phase VO_2_ is distorted in the insulating phase in a monoclinic symmetry with the dimerization of vanadium atoms^7^ as indicated by the empty circles. This dimerization leads to a critical difference between the (101)- and (001)-oriented interfaces; while half of the vanadium atoms cannot dimerize at the (001)-oriented interface as indicated by dashed blue circles in Fig. 8b, all of them can dimerize at the (101)-oriented interface because the dimers can be aligned parallel to the interface as shown in Fig. 8a. This dimerization in the (101) plane was also corroborated by the X-ray diffraction of a 71-nm VO_2_ film on the (101)-oriented Nb:TiO_2_ substrate in Fig. 8c, which shows the (0.5, 0, 0.5) superlattice peak of the VO_2_ film corresponding to the doubling of the VO_2_ unit cell in the plane-normal direction. Thus, the dimerization of interfacial vanadium atoms, which strongly depends on the geometric compatibility of the VO_2_ structural distortion against the TiO_2_ surface orientation, may possibly be responsible for the orientation-dependent current modulation, highlighting that MITs are not purely electronic but are often accompanied by structural transformation. On the other hand, this critical role of interfacial structural compatibility in gate-controlled MITs is to be further studied because the MIT and the structural transformation sometimes take place independently^14^,^45^,^46^,^47^,^48^. Indeed, it is also possible that the MIT by *V*_G_ in our device is not accompanied by structural transformation, and the observed difference between the two interfacial orientations originates from the difference in structural imperfections.

In summary, the solid-state gate-controlled MIT in the ultrathin VO_2_ channel was successfully demonstrated by the high-permittivity TiO_2_ gate dielectric in the inverse-Schottky-gate geometry. This TiO_2_ gate dielectric has a potential advantage compared with the ionic liquid gate suffering from the electrochemical effect and slow response, or the ferroelectric gate^4^,^49^ impaired by a large hysteresis. This solid-state gate dielectric enabled us to discuss the electrostatic mechanism and the collective nature of gate-controlled MITs, which has caused much controversy in the past literature^13^,^14^,^15^,^16^,^17^,^18^,^19^,^20^,^21^,^22^,^23^,^24^. Besides, these results hinted the specific interaction mechanism between the accumulated electrons at the interface and the three-dimensional phase transition, providing the basis for novel device physics incorporating MITs and facilitating the extraction of a variety of functionalities.

## Methods

### Device fabrication

The VO_2_ films were grown by pulsed laser deposition on the (101)- and (001)-oriented Nb:TiO_2_ substrates with 0.05 wt% Nb concentration. The substrate temperature, the oxygen pressure and the laser fluence were set to 300 °C, 1 Pa and 1 J cm^−2^, respectively. All the VO_2_ films had atomically flat surfaces with the root mean square of ∼0.2 nm, except for the film with 28-nm thickness, which showed cracks because of the VO_2_–TiO_2_ lattice mismatch^50^. The VO_2_ films were patterned by diluted aqua regia, and Ag paste was used for the Ohmic contacts to the VO_2_ films. Aluminium wires were bonded for the contact to the Nb:TiO_2_ substrate. Electrical properties of the inverse-Schottky-gate junctions were measured for the VO_2_ film of 9-nm thickness, which is below the critical thickness for crack formation^9^,^44^. For transistors, the thickness was decreased to 6 nm to enhance the gate control, and the source and the drain electrodes were formed with the channel length of 155 μm and the width of 50 μm.

### Electrical measurement

The capacitance was measured by applying an AC voltage of 0.05 V in 500 Hz while sweeping the DC *V*_G_ applied on the Nb:TiO_2_ substrates. The transistor characteristics were measured with the source grounded, and *V*_D_=0.2 V. The temperature was swept from low temperature to high temperature for the heating sweep, reversed to low temperature for the cooling sweep and then *V*_G_ was changed for the next set of sweeps. The transition temperature of the VO_2_ film was defined by the largest negative slope of the logarithmic resistivity against the temperature.

## Additional information

**How to cite this article:** Yajima, T. *et al.* Positive-bias gate-controlled metal–insulator transition in ultrathin VO_2_ channels with TiO_2_ gate dielectrics. *Nat. Commun.* 6:10104 doi: 10.1038/ncomms10104 (2015).

## Supplementary Material

Supplementary Information Supplementary Figures 1-3, Supplementary Notes 1-3 and Supplementary Reference
