# Supplementary material for: Positive-bias gate-controlled metal–insulator transition in ultrathin VO2 channels with TiO2 gate dielectrics
Source: Nat Commun. 2015 Dec 14;6:10104. doi: 10.1038/ncomms10104 (PMC4682056; doi:10.1038/ncomms10104)
Supplement: Supplementary Information — Supplementary Figures 1-3, Supplementary Notes 1-3 and Supplementary Reference [file ncomms10104-s1.pdf]

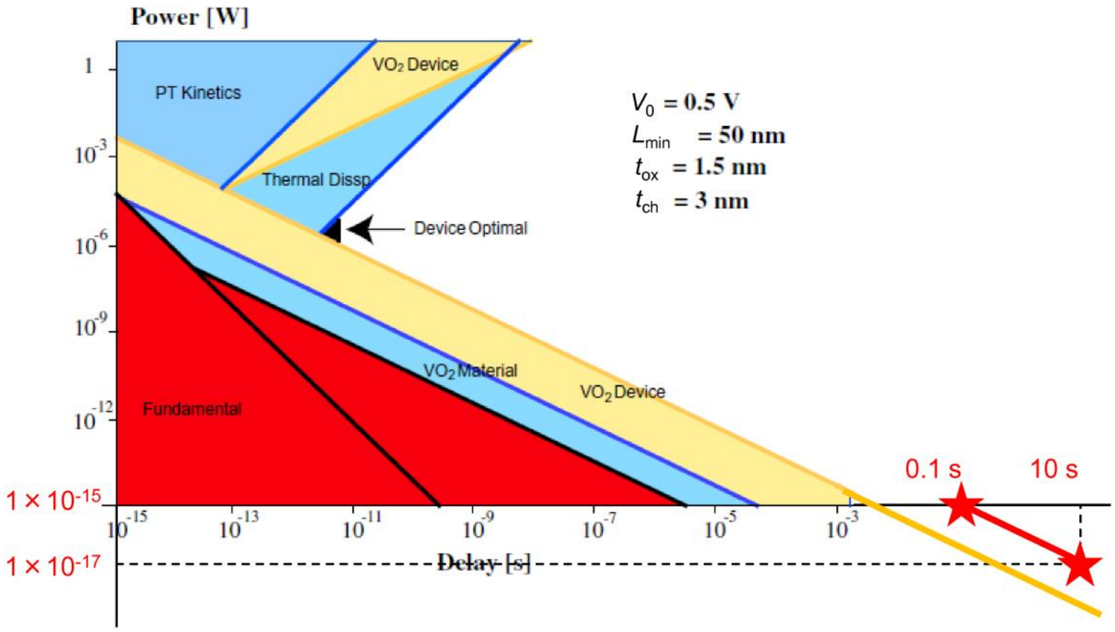

Supplementary Fig. 1: Dynamics of VO_2-channel FETs. Power *vs*. delay diagram of the switching device using VO_2 MIT (cited from ref. 1). The power and delay of our device (normalized by the channel area of 50 × 50 nm^2, which was assumed in ref. 1) are denoted by the red stars.

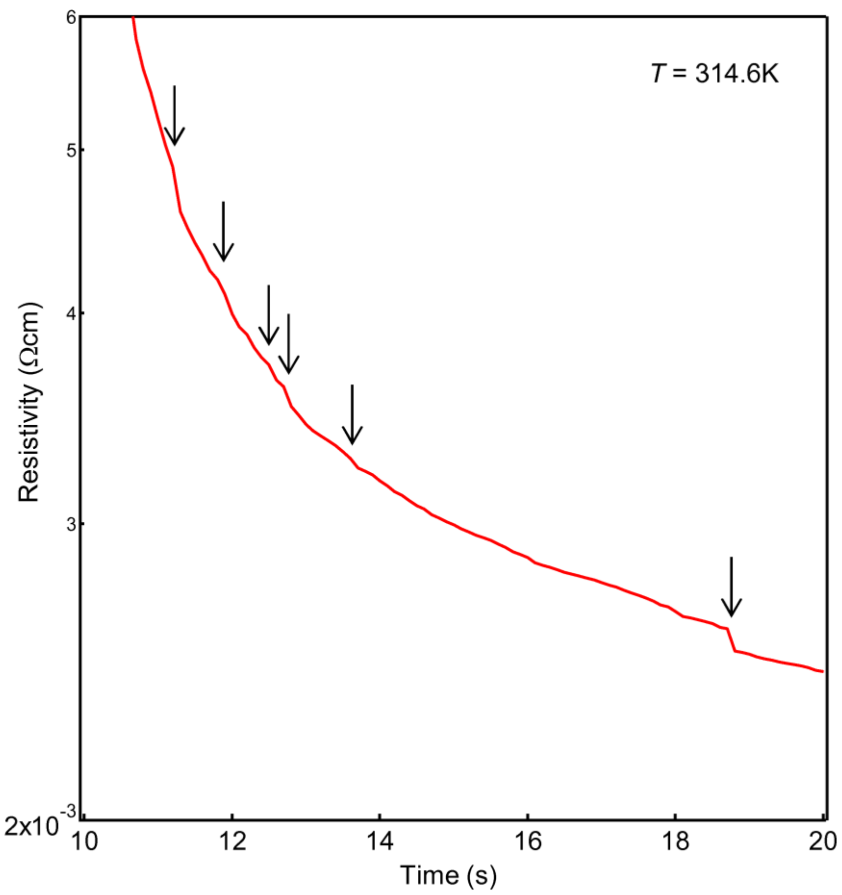

Supplementary Fig. 2: Resistivity jumps in the time dependence. A magnified plot of resistivity *vs*. time in the 6 nm VO_2 channel when V_G = 9 V was applied at 314.6 K (Fig. 5b in the main text). The arrows indicate the abrupt jumps of resistivity.

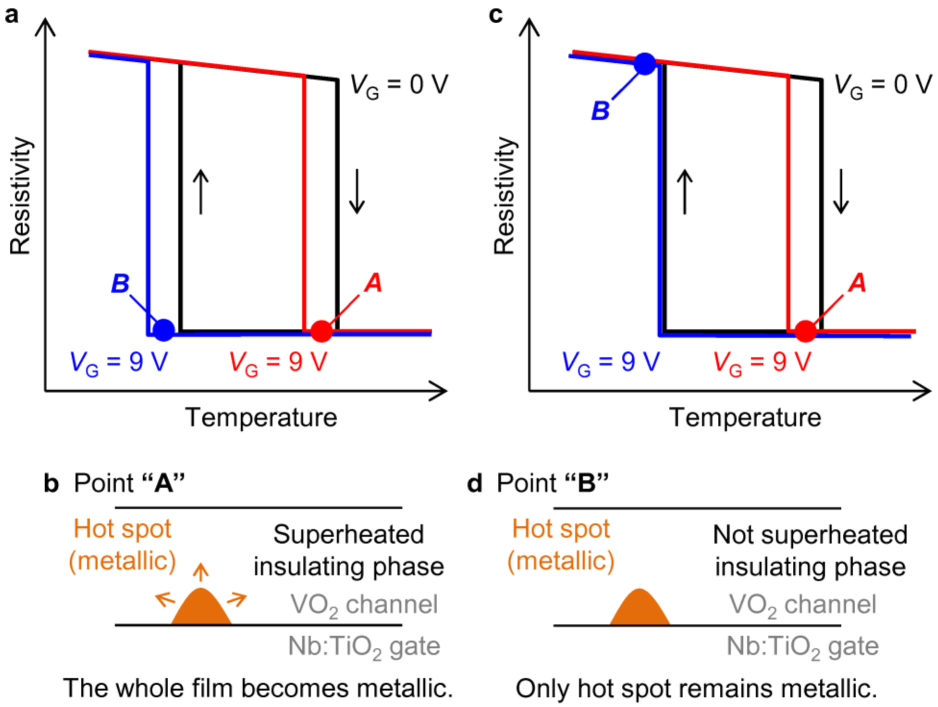

Supplementary Fig. 3: V_G effect via local Joule heating. **a**, **c**, Schematic illustrations of V_G effects on resistivity *vs.* temperature in the VO_2 channel. Black curves correspond to V_G = 0 V and red or blue curves correspond to V_G = 9 V. **a**, A symmetric V_G effect which was observed in our experiments, and **c**, an asymmetric V_G effect via local Joule heating. Point **A** and **B** indicate the states under V_G = 9 V on the high- and low-temperature sides of the hysteresis. **b**, **d**, Schematics of hot spots in the VO_2 channel at Point **A** and **B** in **c**.

Supplementary Note 1: Dynamics of VO_2-channel FETs.

The delay and the power of switching in our VO_2-channel FET were compared with various limits which were discussed previously[1]. We estimated the switching power of our device 1×10^{-15} - 1×10^{-17} W using the delay time scale of 0.1-10 sec (Fig. 5b in the main text), where the channel area of 50 × 50 nm^2 was assumed according to ref. 1. These values are denoted by red stars in the power vs. delay graph in ref. 1 (Supplementary Fig. 1). The values are well within the range where the practical device can physically achieve.

Supplementary Note 2: Resistivity jumps in the time dependence.

When V_G = 9 V is applied, the transition to the metallic phase is induced in the VO_2 channel, and the resistivity decreases with time as shown in Fig. 5b in the main text. When we look at this time dependence data closely (Supplementary Fig. 2), we can see

small but abrupt steps in the evolution of resistivity (arrows), which is the possible signal of phase nucleation and percolation process.

Supplementary Note 3: V_G effect via local Joule heating.

The effect of Joule heating has to be considered carefully because the gate leakage current can be inhomogeneous. The local concentration of gate leakage can raise the local effective temperature, and thermally induce local transition from insulating to metallic phase. And then, it propagates to the whole VO_2 film if it is in a superheated state. Thus, the local effective temperature rise can account for the observed influence of V_G on the high-temperature side of hysteresis (Point "A" in Supplementary Fig. 3a).

At the same time, we can still distinguish the influence of local effective temperature rise by checking the other side of hysteresis (Point "B" in Supplementary Fig. 3a). On the other side of hysteresis, even when a large V_G is applied, the local effective temperature rise by gate leakage cannot stabilize metallic phase in the rest of the film because the rest of the film is not superheated (Supplementary Fig. 3d) in contrast to the superheated state in Supplementary Fig. 3b. Therefore, if the local effective temperature rise is the only origin of the V_G-induced transition in our device, the influence of V_G is expected to be different between high- and low-temperature sides of hysteresis as schematically shown in Supplementary Fig. 3c. Thus, we can distinguish the influence of local effective temperature rise by checking the asymmetry of V_G effect on the hysteresis loop.

Supplementary Reference

[1] Hormoz, S. & Ramanathan, S. Limits on vanadium oxide Mott metal–insulator transition field-effect transistors. *Sol. St. Elec.* **54**, 654-659 (2010).
